# Developments in Fungal Serology

**DOI:** 10.1007/s12281-023-00462-4

**Published:** 2023-04-13

**Authors:** P. Lewis White

**Affiliations:** Public Health Wales Mycology Reference Laboratory and Cardiff University Centre for Trials Research/Division of Infection and Immunity, UHW, Heath Park, Cardiff, CF14 4XW UK

**Keywords:** β-D-Glucan, Galactomannan, *Aspergillus*, *Candida*, Lateral flow assays, Fungal serology

## Abstract

**Purpose of Review:**

The true incidence of fungal disease is hampered by conventionally poor diagnostic tests, limited access to advanced diagnostics, and limited surveillance. The availability of serological testing has been available for over two decades and generally underpins the modern diagnosis of the most common forms of fungal disease. This review will focus on technical developments of serological tests for the diagnosis of fungal disease, describing advances in clinical performance when available.

**Recent Findings:**

Despite their longevity, technical, clinical, and performance limitations remain, and tests specific for fungal pathogens outside the main pathogens are lacking. The availability of LFA and automated systems, capable of running multiple different tests, represents significant developments, but clinical performance data is variable and limited.

**Summary:**

Fungal serology has significantly advanced the diagnosis of the main fungal infections, with LFA availability increasing accessibility to testing. Combination testing has the potential to overcome performance limitations.

## Introduction

Through increased clinical intervention, immunosuppression and modulation, and the impacts of environmental change, the population at risk of fungal disease grows annually, but the true incidence of fungal disease is hampered by conventionally poor diagnostic tests, limited access to advanced diagnostics, and the limited surveillance of most fungal diseases. Serological testing has been available for over two decades in various guises and generally underpins the modern diagnosis of the most common forms of fungal disease, whether acute or chronic in manifestation. While some tests, such as the cryptococcal lateral flow assay (LFA), represent a near-perfect test with high sensitivity and specificity on easily obtained specimens (e.g., serum), other tests (e.g., galactomannan ELISA (GM)) show variable performance, dependent on host and underlying condition, fungal manifestation, and specimen type, yet remain the most established biomarker assay for a particular condition.

This review will focus on technical developments of serological tests for the diagnosis of both acute invasive fungal disease (IFD) and chronic fungal disease but will also describe advances in our understanding of clinical performance when available.

## *Aspergillus* Antigen ELISA Assays

The GM assay manufactured by BioRad likely represents the most well-established/accepted fungal biomarker assay. It has been in clinical use for over two decades, has well-defined positivity thresholds in blood and bronchoalveolar lavage (BAL) fluid, and is recognized as the main mycological criterion for defining invasive aspergillosis (IA) [1]. While the availability of a single assay provides methodological uniformity, it does not guarantee consistent performance, and both sensitivity and specificity of the BioRad GM can fluctuate between centers [2]. A lack of competitive comparator assays minimizes the drive for continued assay development, and so the recent availability of alternative GM is, indeed, encouraging, provided clinical performance is satisfactory (Table 1). When processing large numbers of samples, plate-based ELISA platforms are favorable, but generally limit testing to large centers or reference laboratories. The subsequent logistics of sample transportation and return of results when different laboratory reporting systems are employed can severely hamper utility, even when the testing center provides rapid processing.

**Table 1:** The clinical performance of non-BioRad *Aspergillus* antigen assays

Assay	Sample type	Performance parameter				Reference
		Se (%)	Sp (%)	LR + tive	LR -tive	
Virclia	Serum/BAL	81	100	> 810*	0.19	[3]
Euroimmun	BAL	74	96	18.5	0.27	[4]
	Serum	45	97	15.0	0.57	[5]
	BAL	66	91	7.2	0.38	[6]
	Serum	34	100	> 340*	0.66	
	BAL	89	84	5.6	0.13	[7]
	Serum	43	92	5.4	0.62	
IMMY	Serum	71	98	35.5	0.3	[8]

The availability of GM platforms designed to test small sample numbers has the potential to improve access to testing through wider availability, but much will be dependent on cost and clinical performance. One such platform is the Virclia® Monotest *Aspergillus* Galactomannan AG, a rapid (1 h), fully automated sandwich chemiluminescent immunoassay (CLIA) for the detection of galactomannan in plasma, serum, and BAL fluid. With each monotest containing reagents and controls individual to a sample, a single sample can be tested without using excessive, additional reagents on separate controls. Clinical performance of the test when testing serum and BAL fluid has recently been described (Table 1), concordance with the BioRad GM test was excellent (K: 0.722), with 15 discordant Virclia Positive/BioRad negative being clinical cases of IA compared to three BioRad positive/Virclia negative [3]. The area under the ROC curve using proven/probable IA as a reference was excellent (0.968). The availability of an automated GM ELISA system is useful for minimizing inter-assay variability, where manual operational events could be a potential source of non-reproducible GM positivity and have been attributed to 33% of GM positivity [9]. However, the BioRad GM can be automated using the Evolis system or other widely used systems such as the DS2 Dynex platform, and other non-BioRad automated GM systems are available but lack published clinical validation (e.g., ERA FungiXpert) [3, 10].

The JF-5 antibody utilized in the OLM lateral flow device and detects a galactomannoprotein released from actively growing *Aspergillus* hyphae has recently been incorporated into a plate-based ELISA assay. The Euroimmun *Aspergillus* antigen ELISA generated good concordance with the BioRad GM when testing serum and while sensitivity was poor (45%) when only considering the result from a single, closest to the date of IA diagnosis, it improved (71%) when including assay positivity across a 7-week window [5]. Sensitivity was further improved (96%) using an alternative lower threshold (0.2) derived from ROC analysis, but specificity fell to 76%, compared to 97% when using the manufacturer’s thresholds. Interestingly, when testing serum, the detection of non-hematological cases of IA was potentially better using the Euroimmun assay than the BioRad GM, improving on a known limitation of the latter. Indeed, other non-BioRad GM ELISA assays have been used to achieve an early diagnosis of IA in asthmatic and rheumatology patients [11]. However, a recent evaluation of the BioRad GM ELISA in non-neutropenic patients with a range of *Aspergillus* manifestations (IA, COVID-19 associated aspergillosis (CAPA), chronic pulmonary aspergillosis) generated an overall sensitivity of 89% (range 81–100%) when testing serum and assay specificity was less convincing (75%), primarily compromised by false positivity in patients with histoplasmosis (41%) and tuberculosis (43%) [12]. Conversely, the prospective performance of the BioRad GM ELISA, evaluated by a center proficient at performing this test in a large hematology cohort, generated sensitivity below what would be expected when testing serum (41%) and BAL fluid (78%) [6]. This may reflect the changing status of neutropenia post non-myeloablative allogeneic stem transplantation on test performance, where patients are not as profoundly and prolongedly neutropenic compared to historic myeloablative recipients.

The performance of the Euroimmun assay when testing BAL fluid has also raised questions regarding the suitability of the threshold. Using the manufacturer’s threshold, specificity was excellent (96%), but sensitivity was moderate (74%), although it could be improved (90%) by halving the threshold without compromising specificity [4]. However, in another study evaluating the Euroimmun assay in hematology patients, lowering the threshold when testing BAL fluid did not significantly improve sensitivity (66–75%), but did compromise specificity (91–68%), and the sensitivity was considered inferior to that of the BioRad GM [6]. In this study, lowering the Euroimmun positivity threshold when testing serum did significantly improve sensitivity (34–93%) with only a minor impairment to specificity (100–83%), and the authors concluded that the lower threshold should be considered. Similar findings have been reported in evaluations of other GM-EIA assays (e.g., IMMY GM-EIA) [8].

While well-established in the hematology cohort as the main diagnostic test for IA, the recently described EORTC trial comparing empirical and pre-emptive antifungal strategies in high-risk neutropenic patients not on mould-active prophylaxis confirmed the BioRad GM as a safe method to underpin a pre-emptive antifungal strategy [13]. Overall survival at day 42 was 97% in the pre-emptive compared to 93% in the caspofungin empirical arm, with similar rates of IFD (pre-emptive: 7.7% vs empirical 6.6%) after 84 days. The use of antifungal therapy in the pre-emptive arm was half that in the empirical arm, with no excess mortality or IFD documented.

Another major development in GM ELISA testing was the release of the second revision of the EORTC/MSGERC consensus definitions of IFD [1]. The updated definitions recommend a GM index threshold of 1.0 when testing serum or plasma, BAL fluid, or CSF, double the value recommended by the manufacturer and that previously stated in the first revision of these definitions [14]. While this higher threshold is widely accepted when testing BAL fluid, this increase when testing serum/plasma remains contentious. Justification for this increased stringency when testing serum/plasma was based on the increased likelihood of IA associated with increased specificity (90–94%), while accepting that this could hamper enrolment into clinical trials, it was felt that this improved rigor was critical to underpinning the accuracy of clinical studies. These more stringent classifications were retrospectively applied to 226 cases of proven/probable IA and 139 cases of possible IFD in the *Aspergillus* Technology Consortium (AsteC) and to an antifungal prophylaxis trial [15]. From the AsTec collection, 40 cases of probable IA were reclassified as possible IFD. In the clinical trial, between 31 and 36% of patients were reclassified as possible IFD, and classification of probable IA was delayed by a median of 3 days in 15% of evaluable patients, and there was no difference in mortality in patients retaining a probable classification with those reclassified as possible IFD. Stating a definitive GM positivity threshold in guidelines also has implications when GM ELISA assays other than the BioRad GM are becoming available but potentially with differing units of measurement or different positivity thresholds that reflect the different kinetics of reaction. While a single GM positivity threshold is applied and until there is sufficient available clinical performance data for novel GM assays to be incorporated in guidelines, it becomes critical to understand the correlation in positivity between BioRad and non-BioRad GM assays. The performance of GM assay when testing serum for the diagnosis of *Aspergillus* sinusitis was also recently generated through meta-analysis, providing a moderate sensitivity and specificity of 63% and 65%, respectively [16•].

During the COVID-19 pandemic, GM assays were regularly used to aid in the diagnosis of CAPA, frequently testing samples outside the manufacturer’s current recommendations of serum and BAL fluid. While the GM testing of BAL fluid is widely accepted across patient cohorts and serum positivity in CAPA patients an indication of poor prognosis, performance when testing samples such as sputum, endotracheal aspirates (ETA), and non-directed bronchial lavage (NBL) fluid is unclear [17]. For CAPA, the detection of GM in NBL fluid appears comparable to BAL fluid when using the BioRad GM ELISA or IMMY LFA [18, 19]. For sputum, while GM positivity has been demonstrated in CAPA patients, the diagnostic validity of both positive and negative results is ambiguous, but testing these samples could be advocated in resource-limited settings [20]. For GM testing of ETA, moderate agreement with BAL fluid was demonstrated (kappa: 0.47, accuracy: 80%). Using a higher positivity threshold of ≥ 2.0 for ETA testing generated a BioRad GM sensitivity and specificity of 75% and 81%, respectively [21]. In another study outside of the CAPA population, 92% of GM positive BAL fluids were also GM positive in ETA, but 33 samples were GM positive in ETA alone, indicating that GM negativity in ETA was likely representative of negativity in BAL fluid (98% probability), while GM positivity in ETA was not indicative of BAL GM positivity (40% probability) [22].

## Lateral Flow Assays

Lateral flow assays to aid in the diagnosis of IFD are well established for cryptococcosis and gaining traction for the diagnosis of IA, including CAPA where acceptable performance was attained when testing deep respiratory tract specimens, but has also demonstrated utility for the early diagnosis of CAPA in serum [19, 23, 24, 25]. Their simplicity of use provides rapid sample processing and together with relatively low cost negates the need for batch testing and permits application in resource-limited settings where access to BioRad GM ELISA may be limited [26]. The main issue of subjective, individual interpretation when reading lateral flow strips has been negated by the availability of a digital reader providing quantitative, consistent results.

As with the GM ELISA, cross-reactivity between the IMMY *Aspergillus* LFA and other fungal species (e.g., *Fusarium*, *Scedosporium*, *Candida*, and endemic fungi) has been documented when testing respiratory samples [26, 27]. Clinical performance data is emerging but remains limited when compared to the BioRad GM ELISA, but performance varies dependent on the specific assay used (OLM LFD or IMMY LFA), the specimen tested (BAL fluid or serum/plasma), and the patient cohort. Broadly speaking, the IMMY LFA generally provides good performance when testing BAL fluid or serum, whereas the OLM LFD appears to provide better sensitivity when testing BAL fluid, and in a recent study in hematology patients, the OLM LFD sensitivity when testing serum was poor (26%) [6, 24, 26, 28]. In relation to BAL fluid testing, non-viscous/non-haemolysed BAL fluid can be tested directly using the OLM LFD, permitting point-of-care testing. A recent technical process where neat serum was heated at 120 °C for 15 min prior to centrifugation significantly increased the sensitivity of the OLM LFD to 89%, compared to 56% when performing the manufacturer’s protocol [29]. When compared to the BioRad GM ELISA, the IMMY LFA generated an acceptable, albeit potentially inferior sensitivity (74% versus 89%) when testing serum from non-neutropenic patients with a range of *Aspergillus* manifestations, although specificity was likely superior (84% versus 75%), with IMMY false positivity largely associated in patients with histoplasmosis [12]. The OLM LFD generated poor sensitivity (7%) but excellent specificity (98%) when testing BAL fluid for the diagnosis of chronic pulmonary aspergillosis [30]. Apart from serum and BAL fluid, a lateral flow assay utilizing the capture antibody mAb476 has been developed for the diagnosis of IA when testing urine, and preliminary results are encouraging (sensitivity: 80%; specificity: 92%), with sensitivity varying depending on underlying condition (cancer patient: 90%; other patients: 64%) [31].

An interesting development is the availability of lateral flow assays for the detection of *Aspergillus* IgG and IgM antibodies to aid in the diagnosis of chronic pulmonary aspergillosis (CPA), overcoming some of the limitations encountered in resource-limited settings (Table 2). In an initial retrospective, multicenter French evaluation, sensitivity and specificity were 89% and 96%, respectively, with sensitivity increasing to 91% in a single-center prospective study [32]. Across the range of CPA manifestations, the sensitivity for the diagnosis of CPA (92%), allergic bronchopulmonary aspergillosis (93%, ABPA) was greater than that for invasive/sub-acute aspergillosis (67%), likely reflecting the immune status of patients associated with the latter impacting antibody availability. The antibody LFA was positive in 88% of patients deemed colonized with *Aspergillus*, potentially complicating clinical interpretation. A UK-based study confirmed the excellent sensitivity (92%) and specificity (98%), but also demonstrated that while sensitivity was optimal for the detection of disease caused by *A. fumigatus* (96%), the detection of non-fumigatus *Aspergillus* species remained acceptable (88%) and the LFA was also positive in 89% of cases where sputum culture was negative [33]. Lower specificities (72%) have been documented in some studies and in patients with bronchiectasis in the absence of aspergillosis (82%) [34, 35]. Sensitivity of the LFA for the diagnosis of ABPA and severe asthma with fungal sensitization (SAFS) may be low (< 7%) [36]. A meta-analysis of this assay for the diagnosis of CPA generated pooled sensitivity and specificity of 90% and 91%, respectively, generating positive and negative likelihood ratios of 10 and 0.11, indicating that the test is useful for both confirming and excluding CPA [37]. A recent pilot study compared the performance of the LFA when testing 15 µl of whole blood obtained by a “finger-prick” with that of testing serum/plasma and determined 100% concordance between sample types [38].

**Table 2:** The clinical performance of the LDBio *Aspergillus* ICT lateral flow assay for the detection of *Aspergillus* IgG and IgM antibodies

Manifestation	Population (cases/controls)	Performance parameter				Reference
		Se (%)	Sp (%)	LR + tive	LR -tive	
CPA	154/150	92	98	46.0	0.08	[33]
	44/211	91	96	22.8	0.09	[32]
	262/188	89	96	22.3	0.11	
	20/68	85	72	3.0	0.21	[34]
	74/100	68	81	3.6	0.39	[39]
	30/30	87	90	8.7	0.14	[37]
CPA post TB	20/70	80	70	2.7	0.29	[40]
ABPA	106/164	91	87	7.0	0.10	[35]
	12/374	0	96	< 0.25*	1.04	[36]
SAFS	60/374	7	97	2.3	0.96	

The detection of IgG antibodies against enolase, a metalloenzyme present in the cytoplasm and cell wall of *C. albicans*, has been incorporated into LFA to aid in the diagnosis of invasive candidiasis (IC), generating a sensitivity and specificity of 71% and 96%, respectively, for the diagnosis of candidaemia [41]. During the development of the *Candida* LFA, the authors also developed a plate-based ELISA test which generated a sensitivity and specificity of 87% and 90%, respectively, and concordance between the two tests was excellent (observed agreement: 93%, kappa: 0.851).

The development of an LFA specific to the detection of the principle causes of mucormycosis (*Rhizopus arrhizus* var. *arrhizus* (syn. *Rhizopus oryzae*) and *Rhizopus arrhizus* var. *delemar* (*Rhizopus delemar*)) can help overcome the current lack of a specific antigen test for mucormycosis [42]. Utilizing the IgG1 mAb KC9, which binds to 15 kDa extracellular polysaccharide, the LFA generated a limit of detection of 500 ng/ml in serum and 100 ng/ml in BAL fluid, with no cross-reactivity with other fungal genera, including *Aspergillus* spp., *Candida albicans*, *Cryptococcus neoformans*, *Fusarium* spp., *Scedosporium* spp., and *Lomentospora prolificans* noted. This technical evaluation needs to be followed by a clinical evaluation and with the development of an LFA for the detection of fucomannan in the cell wall of Mucorales species, represents a much-needed diagnostic development for this aggressive disease [43].

## Detection of (1–3)-β-D-Glucan

The detection of (1–3)-β-D-glucan (BDG) in the cell wall of most fungal species is a well-established but not necessarily universally accepted diagnostic strategy. While overall clinical sensitivity and specificity for the detection for generalized IFD is comparable with that of other biomarkers, the lack of fungal genus/species level identification and multiple potential sources of procedural and clinical false positivity can limit the appeal of the test [44, 45, 46, 47, 48, 48]. Nevertheless, the generally high negative predictive value (> 95%) has led to its recommendation for excluding in IFD in national clinical guidelines, underpinning antifungal stewardship schemes [49, 50]. Conversely, BDG positivity is not necessarily indicative of IFD, and the CandiSep randomized control trial investigating whether BDG positivity shortened the time to treatment and improved prognosis in ICU patients at risk of IC presenting with sepsis showed that BDG-guided treatment did not improve survival [51]. As BDG is one of the main fungal antigens recognized by the human innate immunity, generally leading to a pro-inflammatory response and possibly worsening signs of sepsis and mortality, its presence, even in the absence of IFD, may be associated with poor patient outcome, and subsequently, its use to guide antifungal therapy may be misleading, and combining BDG testing with other mycological investigations is warranted [52]. BDG positivity in serum can also be indicative of disease progression and subsequent worse patient prognosis (e.g., CAPA) [17]. Given the multiple sources of BDG false positivity, developing algorithms which incorporate additional mycology alongside BDG testing is critical to gaining an understanding of the probability of infection when presented with multiple, potentially discordant results. Algorithms incorporating BDG alongside PCR have been proposed for the management of PcP and IC, the latter utilizing likelihood ratios to provide the probability of IC according to the various combinations of diagnostic results [53, 54].

The validity and utility of BDG testing of cerebrospinal fluid (CSF) is still to be fully determined, but the presence of BDG in CSF is less likely to be associated with a non-fungal/infective source, and given the range of fungi capable of causing cerebral infection, the application of broad-fungal biomarker could be clinically beneficial. A recent systematic review of BDG testing of CSF included 14 studies describing a range of fungi causing cerebral IFD (*Candida*, *Aspergillus*, *Exserohilum*, *Cryptococcus*, Endemic fungi), and while a meta-analysis was not feasible due to the different causative agents and individual study limitations (e.g., case reports), in general, BDG levels appeared to be elevated in case of cerebral IFD compared to controls, potentially correlating with disease severity and treatment response [55•]. In the five studies where clinical performance validation was possible, sensitivity ranged from 53% for the detection of fungal meningitis caused by *Histoplasma capsulatum* to 100% for *Exserohilum rostratum*, with specificity ranging from 82 to 98%. Interestingly, despite the limited presence of BDG in the cell wall of *Cryptococcus neoformans*, the sensitivity of CSF BDG for the diagnosis of cryptococcal meningitis was 89% [55•]. A recent study demonstrated that the sensitivity of CSF BDG for the diagnosis of cerebral candidiasis and cerebral aspergillosis was 100%, albeit specificity was 70% [56].

BDG positivity has also been incorporated into a risk score model for predicting invasive candidiasis, where multivariate logistic regression analysis identified BDG positivity alongside CD8 + T-cell counts < 143 cells/µl, receipt of high dose corticosteroids, administration of carbapenem/tigecycline, an APACHE II score ≥ 15, and emergency gastrointestinal/hepatobiliary (GH) surgery with a significant risk of IC [57]. Five variables were assigned a weighted score of one point, with GH surgery designated two points based on a larger regression coefficient. Scores of 0–2 were considered low risk if IC, 3–4 moderate risk, and 5–7 high risk. Scores of < 1 had a high negative predictive value (> 98%) for excluding IC; conversely, scores of ≥ 6 had a positive predictive value of 88.9%. The optimal overall threshold was ≥ 3, generating a sensitivity and specificity for the diagnosis of IC of 83% and 68%, respectively [57]. Combining BDG testing with *Candida albicans* germ-tube (CAGTA) antibody (IgG) immunofluorescence generated excellent sensitivity (97%) and moderate specificity (71%) for the diagnosis of IC, when either test was positive [58]. The combined BDG/CAGTA sensitivity was superior for the detection of IC caused by *C. albicans* compared to disease caused by non-*albicans* species. Median BDG concentrations in species other than *C. albicans* associated with candidaemia can be lower (*C. albicans*: 182 pg/ml, *C. parapsilosis*: 78 pg/ml; *C. auris*: 48 pg/ml), leading to a reduction in BDG sensitivity for the diagnosis of candidaemia dependent on species (*C. albicans*: 65% vs *C. auris*: 42%) [59]. The sensitivity of the Wako BDG assay for the detection of IC caused by non-*albicans* species was increased by 24% when the positivity threshold was reduced to 7 pg/ml [60].

The major restriction to widespread accessibility to the test is sufficient sample numbers that subsequently offset the potentially restrictive testing costs. The availability of novel BDG platforms that permit the cost-effective testing of low sample numbers with minimal “hands-on” time is a possible solution (Fuji-Film Wako, Associates of Cape Fungitell STAT, ERA Biology Fully automated Chemiluminescence Immunoassay System (FACIS)), and the availability of multiple, commercially available BDG assays provides competition to control price (Table 3). Currently, clinical validation is limited, or indeed lacking, for some platforms and optimal positivity thresholds still require defining [61, 62, 63, 64, 65, 66, 67, 68, 69]. In general, clinical performance for the non-Fungitell assays is associated with lesser sensitivity than the established test, which can be partially resolved by utilizing positivity thresholds lower than those currently recommended by the manufacturer (Table 3). Some studies demonstrated particularly low sensitivities (50%) for novel BDG tests when diagnosing PcP, a condition typically associated with a high sensitivity (> 90%) [68]. However, this could be associated with the underlying condition of the patient, and pooled BDG sensitivity for the diagnosis of PcP in the HIV-negative patient appears to be lower (86%) [47].

**Table 3:** Recent studies evaluating the performance of various assays for the detection of (1–3)-β-D-glucan in serum

Assay	Population (n/N, (IFD))	Threshold	Performance parameter				Reference
			Se (%)	Sp (%)	LR + tive	LR -tive	
Wako	31/60 (IC, IA, PcP)	7 pg/ml	36	95	7.2	0.67	[67]
	135/187 (IC, IA, PcP)		93	97	31	0.07	[66]
	13/43 (IC)		54	65	1.5	0.71	[65]
	73/34 (IC)		58	85	3.9	0.49	[60]
	129/46 (IC)		70	91	7.8	0.33	
	97/60 (IA/IC)	5 pg/ml	79	88	6.6	0.24	[63]
	120/200 (IC)	3.8 pg/ml	73	91	8.1	0.19	[62]
	63/na (PcP)		95	na	na	na	
	41/188 (IA)	2.4	46	90	4.6	0.6	[69]
Fungitell	28/56 (IC, IA, PcP)	80 pg/ml	61	96	15.3	0.41	[67]
	135/187 (IC, IA, PcP)		98	97	32.7	0.02	[66]
	13/43 (IC)		77	51	1.6	0.45	[65]
	13/28 (IA)		95	51	1.9	0.10	[70]
	97/60 (IA/IC)		92	98	46	0.09	[63]
	120/200 (IC)		87	85	5.8	0.15	[62]
	63/na (PcP)		100	na	na	na	
Dynamiker	23/23 (PcP)	95 pg/ml	87	70	2.9	0.19	[71]
	43/64 (IC, IA, PcP)		81	78	3.7	0.24	[68]
	13/28 (IA)		71	66	2.1	0.44	[70]
STAT	13/43 (IC)	1.2	69	53	1.5	0.58	[65]
	17/49 (CAPA)		71	94	11.8	0.31	[64]
	9/49 (IC)		78	94	13.0	0.23	
	28/49 (IFD)		68	94	11.3	0.34	

## Detection of Antibodies

The performance of assays developed to detect antibodies raised by the host against fungal disease varies dependent on the etiology of the infection, the host’s underlying condition, and the disease manifestation. For chronic pulmonary aspergillosis (CPA), testing for *Aspergillus* specific IgG using commercially available assays is generally highly specific (> 90%) while providing acceptable sensitivity [72]. A range of different methods (*Aspergillus* precipitins, ELISA, Western blot) are available for the detection of IgG, with ImmunoCAP and Immulite assays generally providing superior performance [73]. Across all IgG ELISA assays, the optimal thresholds for the diagnosis of CPA could vary from that defined by the manufacturer and may be influenced by CPA manifestation and the *Aspergillus* species causing infection, given most assays target *A. fumigatus* [73]. The detection of *Aspergillus* specific IgE and total IgE aids in the diagnosis of allergic aspergillois and can be used to differentiate allergic bronchopulmonary aspergillosis from *Aspergillus* sensitized asthma (e.g., IgE against Asp f1-f4), with IgG levels potentially higher in patients with *Aspergillus* bronchitis (e.g., IgG against Asp f1 and f2) [74]. The detection of antibodies in patients with suspected IA is generally limited by the underlying, immunosuppressive condition of the patient, where the risk of IA is associated with the inability to raise a sufficient immune response [75]. However, studies are demonstrating raised baseline levels of IgG against various antigens (HsP90, Pep2, Crf1, and Cdc37) in patients with IA prior to chemotherapy or stem cell transplantation [76].

The detection of anti-mannan IgG/IgM antibodies to aid the diagnosis of IC can be performed using commercial kits from a range of manufacturers (e.g., BioRad, Serion, Dynamiker), but both sensitivity (52–93%) and specificity (54–98%) vary between studies, assays, clinical manifestation, and causative species (optimal detection for *C. albicans*, *C. glabrata*, and *C. tropicalis*) [73]. Combined *Candida* antibody testing (IgG and IgM) or combining antibody testing with antigen testing (BDG or mannan ELISA) or molecular diagnosis may provide improved clinical performance for the diagnosis of IC, but the optimal strategy is yet to be determined [77, 78].

None of the assays described reflect novel technological developments for the detection of fungal antigens. The availability of the previously mentioned CAGTA immunofluorescence assay, with IgG antibodies targeting the hyphal protein (Hwp1) expressed by *C. albicans* during active hyphal growth, may differentiate active infection from colonization, potentially overcoming a known limitation of anti-mannan antibody assays, although the sensitivity and specificity of the CAGTA of the diagnosis of IC varies [79]. A systematic review and meta-analysis of CAGTA performance generated pooled sensitivity and specificity of 66% and 76%, respectively, highlighting the need to combine this test with other mycological assays (e.g., BDG or Mannan ELISA) [58, 79, 80]. The development of LFA for the detection of *Aspergillus* antibodies (described in the lateral flow section) is a significant, recent development in this field, and similar LFA assays for the detection of *Candida* antibodies have been developed. In addition to the Candida LFA utilizing IgG antibodies to target enolase (also described in the lateral flow section), immunochromatographic LFA tests targeting mannan have been developed and are undergoing clinical assessment on a range of clinical samples. Oropharyngeal swabs were tested by ICT to aid in the diagnosis of oral candidiasis generating a sensitivity and specificity of 90% and 91%, respectively, when compared to culture [81]. The development of a dual path platform immunoassay for the detection of *C. albicans* in blood demonstrated a 3.9-fold increase in analytical sensitivity compared to conventional lateral flow modalities, but the reported limit of detection (8 × 10^5^ cells/ml serum) was not encouraging from a clinical perspective, and to date, clinical validation is not available [82]. The availability of FACIS systems with the capacity to automatically perform a range of fungal antigen and antibody tests is encouraging, but clinical validation is currently limited.

New potential targets for the diagnosis of IC have been identified by mass spectrometry-based proteomic analysis of the *C. albicans* hyphal secretome and serum of patients with and without IC [83]. Of the 301 secreted hyphal proteins identified, seven (Bgl2 (1,3-β-glucosyltransferease), Eno1 (Enolase), Pgk1 (phosphoglycerate kinase), Glx3 (glutathione independent glyoxalase), Sap5 (secreted aspartyl proteinase), Pra1 (pH regulated antigen), and Tdh3 (glyceraldehyde-3-phosphate)) were immunogenic with the potential to differentiate patients with IC. The detection of IgG antibodies against enolase has already been reported (see LFA section), and it will be interesting to see if these other potential targets are exploited in the development of future assays [41]. Mass spectrometry has also been used to identify fungal trehalose in the serum of patients to aid the diagnosis of IC, generating positive results in all five candidaemic, BDG-positive patients, whereas mannan antigen ELISA testing was negative in two patients [84].

In relation to alternative targets for aspergillosis antibody detection, an ELISA for the detection of IgG, IgM, and IgA against mitogillin (a ribotoxin responsible for cleaving the phosphodiester bond in eukaryotic 29S rRNA ribosomes) has been described, generating excellent sensitivity (100%) and specificity (95%) for the diagnosis of aspergilloma [85]. Other studies have identified *A. fumigatus* recombinant proteins that were associated with raised antibody levels in patients with aspergilloma and ABPA [86]. While chitosanase CsnB has been studied as a potential antigen for aspergillosis, clinical levels of anti-CsnB IgG antibodies were not useful for the diagnosis of IA [87]. Recent proteome analysis of BAL fluid from a murine model and clinical cases of IA identified 11 proteins that, while not specific for IA, when found in combination may be a useful diagnostic indication of IA [88].

Apart from the main fungal pathogens (*Aspergillus* and *Candida*), the availability of diagnostic antibody tests for other fungal pathogens is limited [89]. Despite the extensive reporting of infections caused by an array of species within the Basidiomycetes division, reports are limited to antibody testing for Schizophyllum commune in patients with allergic bronchopulmonary mycosis and fungal ball [90]. Other antibody tests for potential mould pathogens include IgG against a cell wall mannoprotein of *Talaromyces marneffei* and various antibody assays for the diagnosis of *Pythium insidiosum* infections [91, 92]. The characterization of a fungal aspartic protease allergen Rhi o 1 from the airborne mold *Rhizopus oryzae* could be used to underpin a diagnostic antibody test for mucormycosis caused by this species [93]. Antibody tests for pneumocystosis, cryptococcosis, sporothrichosis, scedosporiosis, and *saccharomyces cerevisiae* infection have been described [73, 94].

## Concluding Remarks

The development of serological tests targeting fungal antigens or strictly targeting antifungal antibodies has greatly enhanced our ability to diagnose fungal disease whether acute and invasive or chronic/allergic in presentation. Despite their availability for over two decades, technical, clinical, and performance limitations remain, and tests specific for fungal pathogens outside the main pathogens are lacking. The availability of LFA to detect both antigen and antibody likely represents a significant development in accessibility to testing in resource-limited centers but can also improve time to result in centers where demand for testing undermines the use of high throughput platforms. The availability of automated systems, capable of running multiple different fungal biomarker and antibody tests, represents a significant technical development, but as with the LFA assays, we are still determining clinical validity and identifying variables that influence performance. The positive impact of serological testing on the diagnosis of fungal disease is obvious, and 65% of diagnostic-driven antifungal stewardship schemes have successfully involved BDG or GM testing, reducing unnecessary antifungal therapy without negatively impacting patient outcome [95••]. Nevertheless, when the performance of serological testing is assessed through systematic review and meta-analysis, it is evident that, CrAg LFA aside, no single test can confidently confirm or exclude IFD and serological testing, while capable of determining refractory fungal disease through persisting positivity, it cannot specifically identify antifungal resistance. Combining tests (whether a combination of different antigens, antigen with antibody, antigen with molecular, or antibody with molecular) appears optimal. For some IFD, such as *Pneumocystis* pneumonia or IA, we may have already identified optimal diagnostic combinations (namely, BDG and PCP PCR and GM and *Aspergillus* PCR, respectively), but this does not mean we should not strive to improve diagnosis further by embracing novel technology (e.g., next-generation sequencing); for other fungal manifestations, we are still to determine optimal diagnostic strategies, and optimal diagnosis of fungal disease in resource-limited settings remains difficult.
